# The cat on a hot tin roof? Critical considerations in multilingual language assessments

**DOI:** 10.4102/sajcd.v66i1.610

**Published:** 2019-05-30

**Authors:** Thandeka Mdlalo, Penelope S. Flack, Robin W. Joubert

**Affiliations:** 1 Livingstone School, Durban, South Africa; 2 Discipline of Speech-Language Pathology, School of Health Sciences, University of KwaZulu-Natal, Durban, South Africa; 3 School of Health Sciences, University of KwaZulu-Natal, Durban, South Africa

**Keywords:** Assessment, cultural and linguistic diversity, English additional language speaker, language dominance, language competence, speech-language pathology

## Abstract

**Background:**

In speech-language pathology (SLP), there is a paucity of resources to provide just and equitable services to South Africa’s culturally and linguistically diverse population. Although South Africa is a multilingual country, English remains the dominant language. However, there is limited research on resources for English additional language (EAL) speakers.

**Objectives:**

This article addresses this gap by presenting the results of a critique of a commonly used language screening tool, the Renfrew Action Picture Test (RAPT), on EAL speakers.

**Method:**

This tool is used as an example to broadly critique the use of culturally biased assessment instruments with EAL speakers from an indigenous linguistic and cultural background. It is administered to children who are EAL speakers and then critiqued by the children too. Their voice, often ignored in research, is central to the research. A mixed methods approach is used, including focus groups and test administration. This article is based on the results of the thematic analysis used to closely examine the patterns that emerge.

**Results:**

A key finding is that the cultural and linguistic background of the child assessed cannot be disregarded, as it plays a crucial role in understanding the response of the child. The interpretation of the response of the child to the presented material of the language assessment tool significantly influences the result of the assessment.

**Conclusion:**

The speech language therapist has a responsibility to avoid skewed results based on uninformed interpretation of the response of the child. These findings provide useful insights for clinicians regarding culture-fair assessment.

## Introduction and literature review

The title, *the cat on a hot tin roof*, draws on a drama, Tennessee William’s drama (Holder, 2016; Williams, 1955), whose existentialist message is relevant to the issues argued. This article presents and discusses the results of a study that critically evaluated and interrogated the use of language assessment tools, in their current form, within the South African context. It also provides the guidelines for adaptations of these tools so that they will better accommodate English additional language (EAL) speakers. The term ‘EAL speaker’ is used in this article to specifically refer to South African multilingual individuals who are non-mother-tongue speakers of English and who are from indigenous language and cultural backgrounds.^1^

The existential message stems from the philosophy that humans are neither mere puppets that exist in the world, nor predestined victims of their conditions in the world. Instead, they are conscious beings who exercise freedom of choice in the decisions that they take in spite of restrictions imposed (Heidegger, 2008). This philosophy is revisited in the ‘*Discussion’* section of the article in relation to the profession speech-language pathology (SLP).

South Africa is a linguistically and culturally diverse country with 11 official languages. The speakers of isiZulu constitute the majority at 22.7% (Stats SA, 2011). Although there are less than 10% English mother-tongue speakers, English is a dominant language in the country. As the profession of SLP is a microcosm of the wider SA society, this dominance is reflected within the profession (Mdlalo, Flack, & Joubert, 2016). The majority of isiZulu mother-tongue speakers are in the province of KwaZulu-Natal, where the research presented was conducted. Although the focus of this article is children who come from the Zulu culture and are isiZulu mother-tongue speakers, issues of culture-fair assessment are broader.

The problem of culture-fair assessment is not unique to South Africa (Laher & Cockcroft, 2017; McLeod, 2014), but a global problem that has been identified in regions such as Australia (Ball & Peltier, 2011), Eastern Europe (Moro, 2008), USA (Hamilton, 2014), New Zealand (Brewer & Andrews, 2016) and Canada (Thordardottir, 2011). However, the suggestion proposed in the study is especially relevant to the South African context as it is based on African language and cultural experience (Makiwane, Nduna, & Khalema, 2016).

The use of standardised tests, with EAL populations, in their current form has been criticised both locally and globally, firstly for linguistic bias, secondly, cultural bias and thirdly, disproportionate representation of different language and cultural groups in normative samples (Caesar & Kohler, 2007; Kathard & Pillay, 2015; Pierce & Williams, 2013). The use of English language assessment tools, in their current form, adversely impacts on the appropriate evaluation of individual clients from other culturally diverse backgrounds, thus preventing them from using their own frame of reference (Moro, 2008; Pascoe & Norman, 2011). These tests are not only inappropriate for use in their current state without adaptation for the South African English mother-tongue speaker, but even more so for the African EAL speaker who is of an indigenous language and cultural background. The speech language therapist (SLT) needs to accommodate the variations in response, which requires an understanding of the South African child’s knowledge and experiences, and the language and vocabulary linked to their daily life and cultural practices (Makiwane et al., 2016). These guidelines and principles could be used by language professionals to manage the assessment process and interpretation of findings from EAL speakers in a more accurate, appropriate and equitable manner.

One of the most common tools SLTs use for language screening, for all clients including African EAL speakers who are from an indigenous language and cultural background, is the Renfrew Action Picture Test (RAPT) (Renfrew, 1997). The RAPT is an expressive screening test for language development that was developed by Catherine Renfrew and originally standardised using English mother-tongue speaking British and Irish children between the ages of 3 and 8 years (Renfrew, 1997). Research findings in the survey, referred to the methodology and reflected in Table 1 revealed that the RAPT is one of the most commonly used screening tests for language development by respondents in this survey in South Africa for all clients, including African EAL speakers who are from an indigenous language and cultural background (Mdlalo et al., 2016). Therapists have remarked that the advantage of using the tool is that it is short, easy to administer and cost-effective (Roulstone et al., 2015). In spite of being aware that the screening tool does not accommodate the indigenous language and culture of the African EAL speaker as it is based on Western^2^ culture and experience, thereby reflecting a Western world view, SLTs commented that their use of it and other tests that are not linguistically and culturally relevant to the SA context is influenced by the paucity of appropriate and culturally relevant resources (Barratt, Khoza-Shangase, & Msimang, 2012; Gxilishe, 2008; McLeod, 2014; Mdlalo et al., 2016).

**TABLE 1:** The research data description and data collection methods.

Phase	Step	Data description and sequence	Method and data collection Tool
Preparatory	-	A descriptive survey via a nationally posted questionnaire. The findings of this phase of the research are covered in a separate article (Mdlalo et al., 2016).	Survey with SLTs Questionnaire
One	Step 1: Test administered	RAPT administered *in English* to all children who met the selection criteria.	Administering of test to children through individual presentation, using language instrument (RAPT), video and audio recording.
	Step 2: Focus group 1	Conducted *in mother-tongue* (i.e. isiZulu) using pictures in RAPT as stimulus for discussion.	Focus group (children) Each group with five to six children Ages 6–8 years From low SES Video and audio recording input.

RAPT, Renfrew Action Picture Test; SLT, speech language therapist; SES, socio-economic status.

As the study focused on the African EAL speaker from an indigenous language and cultural background, research into the child’s indigenous knowledge system is crucial to ensure that any proposed adaptation is relevant to the daily experiences of the child, and will provide essential information that reflects the child’s indigenous world view (Chivaura, 2006). Failure to ensure this could result in an assumption of a language problem which does not exist, and lead to inappropriate recommendations or even intervention (Cofresi & Gorman, 2004).

## Methodology

The aims of the study were to establish the implications of using a currently favoured language screening tool in South Africa, the linguistic and cultural relevance of this screening tool for isiZulu-speaking children as an example, and how the language screening assessment tool could be adapted to be more linguistically and culturally relevant.

A qualitative approach was used to determine the perspectives of the various groups of participants and to analyse the findings. This provided a relevant framework to illustrate how people drew in-depth meanings from their social actions, background, culture and world view, and how these responses could be interpreted, comprehended and appreciated.

The RAPT served multiple methodological purposes in this study, as reflected in phase 1 in Table 1. It was used for individual testing of the children to demonstrate differences in interpretation and as an example for discussion in the focus groups with the children.

The article mostly focuses on phase 1, where the participants were 90 children (see Table 1).

### Step 1: Individual testing

Although the administration and scoring manual of the research instrument required that it be scored quantitatively (Renfrew, 1997), in this particular research, it was used qualitatively as it was not used to identify language disorders per se, but was used as a ‘case study’ to demonstrate the effects of culture and different language upon interpretation of test stimuli. Because it is one of the most commonly used language screening tools in South Africa, the RAPT was used in this study to determine whether there were consistent deviations in the responses from EAL isiZulu-speaking children to the questions on the various picture stimuli. It was applied to the children in the study who were from low socio-economic backgrounds and whose ages ranged from 6 to 8 years exactly as stipulated in Table 1 and the responses were recorded verbatim, as indicated in the test administration manual. A total of 50 children were used for the test administration. These were then interpreted and analysed qualitatively.

Although the cultural and linguistic relevance of this commonly used screening tool was interrogated from four different viewpoints, this article focuses on the perspective of the children who are the target population of the tool. As language assessments are conducted with people who exist within a cultural context, the cultural capital is embedded in language (Blair, 2016; Espinoza-Herold & González-Carried, 2017; Haneda, 2014; Smith, 2015; Westby, 2009).

This study focused on the relationship between language and culture and adopts an ecological approach to the problem; thus, a conceptual model that encompasses a strong ecological and cultural component was selected, that is, Taylor’s (1986) cultural framework for viewing normal and pathological communication.

### Step 2: Children’s focus group

A total of 40 children from the isiZulu linguistic and cultural background were divided into focus groups consisting of five to six pupils in each group. Prior to the actual testing, individual test administration piloting was conducted. Piloting of both the test administration and the ‘voice of the children’ group was beneficial as it acted as a dress rehearsal for the actual test administration and also provided an opportunity to review data collection techniques (Leedy & Ormrod, 2010). The children’s focus group was piloted using a group of 10 children consisting of equal numbers of boys and girls. Problems that were identified were addressed prior to the individual trial-testing and the children’s focus group sessions. These included improving the clarity of the questions, ensuring that sufficient rapport was established prior to the individual and group sessions and further clarification regarding which language the children should use in their responses was required.

### Profile of the children in the study

Black, isiZulu mother-tongue speaking children from both urban and rural schools participated in the study. The urban schools selected were ones that are categorised as ‘no-fee’ paying by government as the majority of the children came from low-income homes (Burger, 2011), and were the children who went to the farm or rural areas during the holidays. Their dual experiences, of both urban and rural environments, were reflected in their responses. In this study, only the urban school was English medium, while the rural schools were isiZulu medium. The children in rural schools were only exposed to English at school or via the media.

As the test was used to screen children, it was integral to this study that the voice of the children be heard (Messiou, 2013). The soliciting of the voice of the children was performed through individual sessions and focus groups. Table 1 summarises the data collection methods and data analysis strategies of the full study.

## Ethical consideration

Ethical clearance for the research (Ethical approval number HSS/115/100) (see Appendix 1) was provided by the University of KwaZulu-Natal Humanities and Social Sciences Research Ethics Committee.

## Results

The findings of the study, that is, individual children assessment and focus groups, were analysed and the emerging patterns discussed. The responses were reviewed, and based on the emerging patterns, the implications these findings had for the speech-language pathology (SLP) profession were explored.

The format used to depict the results was to present each picture from the Renfrew Action Picture (RAPT) and under each picture, the responses from each of the participants. The response was followed by a commentary. The visual stimuli^3^ and responses were categorised into the themes depicted in Table 2.

**TABLE 2:** A table outlining the content of visual stimuli in the Renfrew Action Picture Test.

Themes	Visual stimuli in RAPT^©^ (Speechmark Ltd)
5.1: Human relations and respect	Girl hugging a teddy bear – Picture 1 Kneeling woman helping child – Picture 2 Girl falling down the stairs – Picture 6 Baby lifted by older girl – Picture 7 Boy picking up fallen apples – Picture 10
5.2–3: Perception of animals	Dog tied to a pole – Picture 3 Man riding a horse – Picture 4 Dog with a crying boy – Picture 9 Cat with mice – Picture 5 Cat on the roof of a house – Picture 8

RAPT, Renfrew Action Picture Test.

Interpretation of the patterns emerging and support for the assertions about the African culture and experience were drawn from the literature of various disciplines such as anthropological, psychological, linguistic and sociological writings (Kunnie & Goduka, 2006; Okon, 2013) that are related to the themes emerging in the study. The black African culture, stories, philosophy and traditions have a formidable oral tradition that is reflected in, or is the underlying reason for, some of the practices that may be perceived as different, irrational, unreasonable, purposeless or even primitive when judged and viewed through the Western^4^ lens (Higgs, 2010; Mucina, 2013). Some of the actions or interpretations of the pictures of the participants in the study are embedded in this oral tradition and there may be a paucity of literature on them, for example hugging in black African culture. This paucity of literature presents with numerous difficulties and constraints in the interpretation and translation of the responses because the interpretation of the responses is influenced by language as ‘ideas and philosophies created in one language cannot always be adequately translated into another language without losing some meaning because each language speaks to a specific contextual symbolic encoding’ (Mucina, 2013, p. 24) because of language translational limitation (Barratt et al., 2012). Mucina’s remark illustrates how the process of translating or interpreting across languages can present a challenge to accurate symbolic expression and cultural transmission of ideas and philosophies. When translating, there is an attempt to impose a certain level of resemblance and equivalence in word or concept meaning. However, the interpretation of the word or concept is influenced by factors such as culture, world view and frame of reference of the speaker. The result is what may be regarded as a logical deduction, but may not be relevant, as the mental representations differ despite the seeming resemblance and coincidences in meaning. An example is the root or stem in isiZulu, – *nde*, encompassing both the meanings and associations linked to the words *tall* and *long* in English and often presents with many misinterpretations in conversations when directly translated. Another example is use of the word ‘checkers’ to refer to the bag (in picture card 10). In townships, any grocery plastic bag is commonly referred to as a ‘checkers’. These examples show that words that have a meaning that may be clear for an English First Language (L1) speaker may not necessarily have the same meaning from the perspective of an L2 speaker. They may be ambiguous based on how the L2 speaker has experienced their being used, that is, interlingual ambiguity.

These restrictions manifest in the analysis of the responses. For example, the use of clan names between communicators may represent the same level of intimacy as hugging and yet, despite the seeming resemblance to nicknames in Western culture, not have the same mental representation or meaning.

**FIGURE 1:**
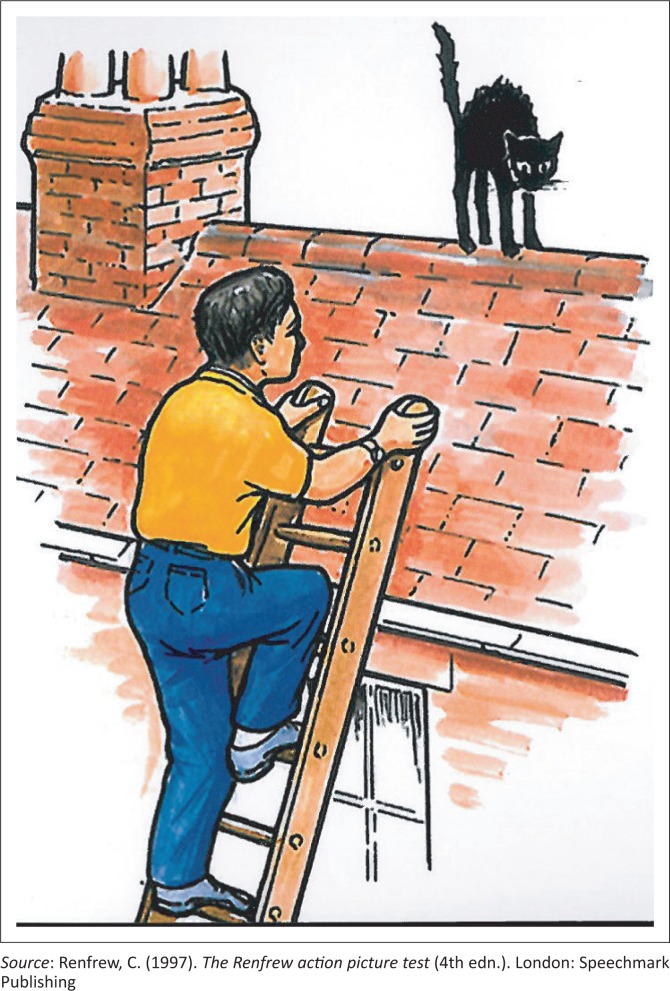
Illustrating picture card 8 from the RAPT (1997) (^©^Speechmark Ltd).

Picture card 8 on the RAPT (see Figure 1) has been selected in this article to illustrate the argument, as it was unanimously the most controversial picture. The controversy was around the black cat and how it is perceived in the black community (Mutwa, 2003). A black cat is usually associated with witchcraft and negativity. As a pet, it is to protect the home by killing unwanted pests, such as snakes and mice (Mutwa, 2003). The concept of rescuing a black cat was regarded by most focus group participants as inviting danger. The responses from the children (see Tables 3 and 4) clearly indicated that the security of the man climbing the ladder was at stake. Therefore, in the following phase of the study, the picture was adapted to be more culturally appropriate and the black cat on the roof was replaced by a ball that was to be ‘fetched’ from the roof of the house (Mdlalo, 2013).

**TABLE 3:** Some results of the voice of the children for each picture.

Picture	Summary of children’s responses	Relevance for SLT or themes emerging
1	Referred to the toy as *teddy bear or doll*; did not mention hugging but rather quieting and comforting the baby ‘*shushuzela*’. Rural children frequently mentioned racial differences.	Differences in perception of demonstration of intimacy, for example hugging. Despite the universality of concept of play, the form it takes in different communities and culture differs.
2	Highlighted the mother kneeling; helping of a child to dress associated with a younger child. It is culturally inappropriate for an adult to kneel for a child in the Zulu culture.	Differences in perception of child–child versus child–adult relations or roles, reflected in responses.
3	They perceived the dog as for protection. It was seen as a pet but they clarified that it was not to be stroked or stay in the house.	Differences in perception of roles of animals reflect.
4	Rural children could not relate to the horse as depicted in sport in the picture, did not know the concept of a jockey or saddle; saw it as a useful animal that can save one from danger as used in own context.	Exposure affects responses.

SLT, speech language therapist.

**TABLE 4:** Children’s responses and reasons for adaptation of the Renfrew Action Picture Test expected responses.

Expected response in RAPT	Actual response of children	Reasons for adaptation
Climb up or on, go up to, try to go up, ladder, get, fetch, reach, save, rescue, cat, kitten, pussy, on or off, down from or off, from roof, top of house	Climb up or on, go up, try to go up, ladder, get, cat on or off, from roof, roof, chase, fix, step or stairs, take, catch, run away, man, get, catch, father, boy, up, cat, top	Words not used were ‘reach’, ‘save’, ‘rescue’ and ‘pussy’. ‘save’ and ‘rescue’ are linked to the perception of the cat as being in danger South African English, the word ‘pussy’ is not usually used in isolation Although ‘ladder’ is a commonly used word in English, colloquially, African language speakers have often replaced it with the word ‘steps’

RAPT, Renfrew Action Picture Test.

It was interesting that the concepts of witchcraft and traditional African values are usually mostly associated with the rural areas (Durrheim, Mtose, & Brown, 2011), and yet it was the urban children who did not like the picture because of these associations. The rural children’s fear of a cat was similar to their fear of any other potentially dangerous animal and was not for culturally related reasons, whereas urban children’s aversion to the black cat as a dangerous animal was more reflective of the cultural view. This response suggests that assumptions that are often associated with the distinction between urban and rural communities are not always accurate. This finding shows that speech-language therapists (SLTs) should be cautious of using typical stereotypes pertaining to urban and rural communities (Durrheim et al., 2011).

The responses of the children illustrate that verbal associations with the picture or words may be different from those expected by the SLT, as associations are linked to experience, background and culture. Verbal associations are thus linked to sociolinguistic factors.

The responses from the children were often characterised by pregnant pauses or slow responses. The detection of the words in the question and planning a response based on the detected word is a complex process when the question is not in the L1. This slowness in word detection may be misinterpreted as word-retrieval or word-finding problems. De Rauville (2004) argues that in EAL speakers, certain behaviours may seem to be word-finding errors, such as word-searching, whereas these errors may be a reflection of their limited semantic knowledge in the second language (De Rauville, 2004, p.17) and therefore not a language pathology.

## Discussion

The responses from the children accentuate numerous critical issues that the SLT needs to be aware of in working with culturally diverse populations.

Apart from the theory, the philosophical lens through which the SLT views and interprets this information will determine how it is used and whether justice is served (Arias & Friberg, 2017; Fricker, 2007; Morreira, 2017). If the SLT uses his or her own frame of reference to interpret, the picture presented may still be skewed. Often, the perception of the dominant culture is used as a basis for whether it is pathological or not. In South Africa, the Western culture of the English L1 speaker is often part of this dominant culture and Western standards become the basis and bias for the decision as to whether a communication difference is a communication difficulty or difference. The examples above of children’s utterances from pictures cards 4 and 10 provide an illustration of linguistic differences that may be misconstrued as part of a syntactic disorder but influenced by the L1 (Table 5).

**TABLE 5:** Linguistic differences that may be misconstrued as part of a syntactic disorder.

Utterance	Explanation
The man, he rides a horse	The isiZulu concordial structure ***u***muntu ***u***gibela superimposed
The lady, he walks	There are no gender grammatical structures in isiZulu

Language transfer occurs when the characteristics of one language affect those of another. These may occur at a morphological, syntactic, semantic or pragmatic level (Myers-Scotton, 2006). An understanding of the structure of isiZulu as a language will help to make sense of these language differences, which may be depicted as errors because of misinterpretation or misunderstanding

Another illustration from the picture card 8 is the use of the word *ladder*. Although the direct translation of the word is *íleli,* in practice it is commonly used interchangeably with *amastepisi* (steps) and this was reflected in the children’s responses. An SLT who has no understanding of this frame of reference will regard the *steps* as inaccurate.

Based on the existentialist message in the *introduction*, SLTs are free to choose to use the client’s frame of reference despite the challenges of limited knowledge and resources that may exist. Cultural sensitivity will help the SLT to understand the responses and interpretation of responses that may seem inappropriate or irrelevant.

Within the profession, both globally and locally, there is consensus on the need to transform the current paucity of material and guidelines on the assessment and management of culturally diverse populations. Research, assessment tools and profiles have been developed by professionals and tertiary institutions in an attempt to bridge this gap (Pascoe & McLeod, 2016; Singh et al., 2015; Van der Merwe & Roux, 2014). There is, however, an acknowledgement that, despite these attempts, what has been produced to resolve this social reality is a drop in the ocean in terms of the needs within the profession (Khoza-Shangase & Mophosho, 2018).

Anecdotal evidence suggests that there is a practice that, despite the extremely limited resources available for assessment of the EAL speaker, the tools that exist, for example South African Language Assessment (SALA – in all South African languages) and Test of Ability to Explain for Zulu Speaking Children (TATE-ZC), are not used. There has been very little research that has tapped into this issue in South Africa. In this study, some of the changes made to the research instrument, the RAPT, based on the responses of the participants, have already been made to other tests (Solarsh, 2001; Solarsh & Alant, 2006), but these changes appear not to have been implemented in the use of these tests. They are still used in their original form, not taking into account the research-based adaptations for multicultural clients. The question that arises is whether SLTs are guilty of what critical theorists would describe as ‘social oppression’ by ignoring more culturally and linguistically appropriate resources for assessment. If the answer is positive, the critical theorists (Han & Price, 2015) propose a process of assisting them to become aware of the manner in which this social oppression operates through the process of conscientisation, that is, a process of creating awareness of another social reality, thereby helping to free them from social oppression.

A critical theorist, drawn from the field of education, who advances this argument competently, is Paulo Freire. The process of conscientisation is also central to his educational theory of radical social change (Freire, 2000), which focuses on the root causes of social and political problems rather than the symptoms, to allow for the planning of strategies to address them. Freire argues that critical consciousness is essential to reassess the types of ideas, contexts and relationships that are typically ‘taken for granted’ or accepted as the norm prior to ascertaining the root causes of social oppression (Freire, 2000). Furthermore, he argues that authentic engagement with people you are committed to requires constant self-reflection.

Based on Freire’s argument, a question that arises is whether SLTs are constantly self-reflecting on how to best engage with the challenge of EAL speakers or are complacent with the current scenario of the scarcity of appropriate and culturally relevant assessment instruments for EAL speakers from indigenous linguistic and cultural backgrounds. Freire regards such a scenario as reflecting a degree of complicity. This means that SLTs may be aware of the gaps or contradictions but choose to turn a blind eye to them.

From an SLT perspective, we argue that conscientisation forms part of the solution. We believe that this process is long overdue and would play a significant role in addressing the social injustices of South Africa’s past (Kathard & Pillay, 2013). The continued use of tests on EAL speaking children that are normed on predominantly English-speaking UK or USA populations in their current form can present the SLT with inaccurate findings that may be used to determine the future of the children assessed (Arias & Friberg, 2017; Williams & McLeod, 2012). The future of these children therefore rests on the use of the RAPT and any other language assessment test in a culturally and linguistically relevant manner. Furthermore, the SLT will be able to conduct a comprehensive assessment and make an informed decision based on a holistic picture of the child’s competencies. The SLP profession has a responsibility to conscientise itself (Kathard & Pillay, 2013) in a culturally and linguistically relevant manner regarding the use of the currently available tools. Practically, this entails SLTs understanding the language or culture of the client, understanding bilingualism and second language acquisition.

## Conclusion

The results of the study show that the heterogeneous nature of the South African population, especially pertaining to language and culture, places certain responsibilities on the SLT to ensure ethical, just and moral assessments. It is pivotal to acknowledge that heightened consciousness and reflection form part of this process. As each SLT is an intentional being (Heidegger, 2008), this process cannot be a tacit one, but should be conscious and active as it has implications for the extent to which the SLP profession can continue to engage in transformation that will result in just and equitable service provision for all the people of the country irrespective of colour, race, creed, culture, religion or gender (Brewer & Andrews, 2016; Espinoza-Herold & González-Carried, 2017). A collaborative effort by private, public and tertiary institutions is crucial for the success of this transformation. Despite the intimidating nature of the process, we do not have the option to surrender and allow frustration to dictate the direction of transformation. As SLTs, we dare not to define ourselves by our external circumstance as we are not at the mercy of the limitations linked to diversity but can collaboratively, consciously and reflexively explore the alternatives as we celebrate this diversity.

## Footnotes

1 Not a homogenous group, there may be inter- and intra-linguistic and cultural variability.

2 Despite the risk of overgeneralising as the Western culture is not composed of a single, homogeneous set of values, the term ‘Western’ has been used as reflected in the literature (Miller, 2012).

3 The visual stimuli from the RAPT, fourth edition published in 1997 and reprinted in 2010, were used in the study. The 2010 publication (accessed at the end of the writing of the study) of the RAPT has revised visual stimuli. The content of the visual stimuli has been modernised and also reflects different races. These revisions are noted and acknowledged but do not change the findings and central arguments in this study.

4 Variations in both interpretation and practice of African culture.
